# Kinetics of tissue oxygenation index during fast and slow cardiopulmonary bypass initiation

**DOI:** 10.1177/02676591211068972

**Published:** 2022-01-25

**Authors:** Jan Turra, Adrian Bauer, Andreas Möbius, Jacob Wojdyla, Christoph Eisner

**Affiliations:** 1 Department of Cardiothoracic Surgery, 27178 University Hospital Heidelberg, Heidelberg, Germany; 2 Department of Cardiovascular Surgery, Mediclin Heartcenter Coswig, Coswig, Germany; 3 Department of Cardiothoracic Surgery, 23536 Sunnyside Medical Center, Oregon, USA; 4 Department of Anesthesiology, 27178 University Hospital Heidelberg, Heidelberg, Germany

**Keywords:** near infrared spectroscopy, cardiopulmonary bypass, initiation time, cerebral oximetry

## Abstract

**Introduction:**

Despite being a daily clinical application in cardiac operating theaters, an evidence-based approach on how to optimally initiate the heart–lung machine (HLM) to prevent critical phases of cerebral ischemia is still lacking. We therefore designed a study comparing two different initiation times for starting the cardiopulmonary bypass (CPB).

**Methods:**

We conducted a monocentric, randomized, and prospective study comparing the impact of two initiation times, a rapid initiation of 15 s and a slow initiation of 180 s to reach the full target flow rate of 2.5 L/min/m^2^ times the body surface area, on cerebral tissue oxygenation by near infrared spectroscopy measurements.

**Results:**

The absolute values in tissue oxygenation index (TOI) showed no difference between the groups before and after the CPB with a 10% drop in oxygenation index in both groups due to the hemodilution through the HLM priming. Looking at the kinetics a rapid initiation of CPB produced a higher negative rate of change in TOI with a total of 21% in critical oxygenation readings compared to 6% in the slow initiation group.

**Conclusion:**

In order to avoid critical phases of cerebral ischemia during the initiation of CPB for cardiac procedures, we propose an initiation time of at least 90 s to reach the 100% of target flow rate of the HLM.

## Introduction

Concerning the initiation of cardiopulmonary bypass (CPB) using the heart–lung machine (HLM), there is currently no well-described recommendation by the manufacturer or by scientific evidence for the safest speed or time frame to reach the desired cardiac output (CO) of the HLM. Around 30 years ago, it was described that a full CO with the HLM can be achieved in 30 s in most cases.^1^ This information has since continued to be taught without scientific evidence, and initiation times vary widely by institution ranging from 10 s to 20 s or up to 300 s in specific cases (e.g. aortic dissection).^2^ There are theoretical neurological benefits to slower initiation times. In contrast to a fast initiation, a slower ramp-up time causes softer hemodilution following better endogenous compensation of the decreasing oxygen supply and thus possibly causes fewer neurological complications. This study compared the impact of two initiation times, a rapid initiation of 15 s and a slow initiation of 180 s, on cerebral tissue oxygenation index (TOI) measurements and looked for critical readings of TOI indicating cerebral ischemia.

## Methods

We conducted a monocentric, randomized, and prospective study at the Heidelberg University Hospital for Cardiac Surgery from October 2017 to July 2018. Our institutional ethics review committee approved the study protocol (file number S-410/2017) on 28th. September 2017, and written informed consent was obtained by all participants. Inclusion criteria included adult patients (gender independent) over 18 years of age scheduled for non-emergent coronary artery bypass graft (CABG) surgery with arterial cannulation of the aorta and single venous cannulation of the right atrium. Exclusion criteria included history of neurological disease (e.g. stroke), stenosis of the carotid or cerebral arteries, noticeable calcification of the aorta, anemia defined as a hemoglobin level lower than 10 g/dl or a hematocrit level below 30%, as well as patients in need of a preoperative cardiac support system (e.g. intraaortic balloon pump, extracorporeal membrane oxygenation or ventricular assist devices). Baseline demographic and medical data were collected for all patients. Before the study, a block randomization was performed (BiAS. for Windows™, version 11.10, epsilon-Verlag GbR Hochheim Darmstadt, Germany) for groups A and B and defined as followed:

A. Cardiopulmonary bypass initiation time of 180 s to reach 100% of the target flow rate.
B. Cardiopulmonary bypass initiation time of 15 s to reach 100% of the target flow rate.

The target flow rate was set at the calculated CO of each individual patient according to a cardiac index of 2.5 L/min/m^2^ multiplied by the body surface area formula after DuBois and DuBois. Blood volume was calculated with a factor of 65 mL per kg bodyweight in female patients and 75 mL per kg bodyweight in male patients.

### Study protocol—NIRS, anesthesia and surgery

Tissue oxygenation index (TOI, %) and tissue hemoglobin index (nTHI, arbitrary unit) levels were measured using near infrared spectroscopy (NIRO-200NX, Hamamatsu Photonics K.K., Hamamatsu City, Japan) by having the respective electrodes placed on the left and right forehead, starting with awake measurements before anesthetic induction. For technical and mathematical insight of the NIRS measurement by Hamamatsu Photonics, kindly refer to available literature.^3–7^ All patients were anesthetized to the same standard protocol. Anesthetic perioperative management consisted of arterial and venous line placements, transesophageal echocardiography monitoring and administration of norepinephrine, and saline infusions to keep the mean arterial pressure between 50 and 80 mmHg. All patients received a midline sternotomy and the same cannulation procedure for the CPB. Complete heparinization was achieved with 400 IU/kg bodyweight of heparin (ACT > 450 s) prior cannulation. The return cannula (24 Fr) was placed in the distal ascending aorta and the two-stage drainage cannula (34 Fr) was placed over the right atrial appendage into the inferior cava vein. An aortic root cannula (9 Fr) was placed in the ascending aorta to give the cardioplegia on the first port and on the second port, we connected the aortic vent. The return cannula was connected to the arterial line (3/8 x 3/32-inch tubing) and the drainage cannula was connected to the venous line (1/2 x 3/32-inch tubing) of the HLM.

### Study protocol—HLM

The set-up of our HLM was as follows: we used a LivaNova S5 (LivaNova PLC, London, United Kingdom) HLM as well as a Sorin Inspire 6F (LivaNova PLC, London, United Kingdom) oxygenator module with integrated arterial filter and open hard-shell venous reservoir. The priming of the HLM circuit was carried out with 1000 mL of balanced electrolyte solution (Sterofundin^®^ ISO, B. Braun Melsungen AG, Germany) including 10.000 IU of heparin. Before initiation of extracorporeal circulation, the priming solution was preheated to 37°C and the F_i_O_2_ was set at 0.6 at a gas flow of 2.0 L/min. After baseline recordings, the CPB was started according to the group specifications. In Group A, the flow was increased in a stepwise manner every 45 s by 25% of total flow to reach the target flow of 100% at 180 s. Likewise, in Group B, the flow was increased in a stepwise manner every 3.75 s by 25% of total flow to reach the target flow of 100% at 15 s. The venous clamp was closed to 10% to reduce the diameter of the venous tube before the clamp on the arterial side was removed. The clamp of the venous side was then gently removed in such a way to ensure that the patient stayed isovolemic during the initiation of the CPB and therefore pulsatility was maintained throughout the measurement period. The target mean arterial pressure was kept between 50-80 mmHg and if necessary supportive norepinephrine or nitroglycerine doses were allowed. The extracorporeal circuit temperature of the blood remained at 37°C for the time of the measurement. After the CPB reached the individual target flow rate of 100%, the surgeon and anesthesiologist carried on with the surgery as planned after an additional 60s equilibrium measurement period.

### Statistical analysis

Data from the near infrared spectrometer were recorded at 2 Hz and data from the heart lung machine were recorded at 0.05 Hz. After conversion with the company’s own Hamamatsu software, the patients’ NIRS data were combined into an Excel file and supplemented with data on perfusion and the patient’s characteristics. Mean values and standard errors were calculated for all data. Means were examined for a significant difference using the two-sample t-test for independent and dependent variables. A *p*-value less than .05 was considered to be statistically significant. Each respective data set of TOI readings was screened for critical readings of under 50%, a decline of over 20% and 80% of baseline. The statistical processing and graphical presentation of the results was done with the Graphpad Prism5 software.

## Results

Patients’ characteristics, target flow rate and blood dilution through the CPB circuit are shown in Table 1. There were no differences in age, height, bodyweight or calculated target flow rate and blood volume. Group B had lower hemoglobin and hematocrit values before (pre-bypass) and after the NIRS measurement period (post-bypass initiation) than Group A (*p* < .05). None of the groups needed vasoactive drugs for keeping the target mean arterial pressure during the initiation period.

**Table 1.** Baseline characteristics and pre- and post-bypass initiation hematocrit and hemoglobin levels in Group A and B. Data values are means ± standard error. *p*-value either not significant (ns) or significant (< .05).

patient characteristics		Group A (n = 31)	Group B (n = 29)	*p*
age		68 ± 2	68 ± 2 years	**ns**
height	meas.	173.5 ± 1.2	172.2 ± 1.3 cm	**ns**
bodyweight	meas.	84.6 ± 2.5	80.7 ± 2.5 kg	**ns**
BMI	calc.	28.2 ± 0.9	27.1 ± 0.7 kg/m^2^	**ns**
BSA	calc.	1.98 ± 0.03	1.93 ± 0.03 m^2^	**ns**
target flow rate CPB	calc.	4.97 ± 0.07	4.83 ± 0.08 l/min	**ns**
blood volume	calc.	6.3 ± 0.2	6.0 ± 0.8 l	**ns**
hematocrit	pre-bypass meas.	41.6 ± 0.6	39.6 ± 0.8 %	**<.05**
	post-bypass initiation meas.	31.9 ± 0.6	29.2 ± 0.8 %	**<.05**
hemoglobin	pre-bypass meas.	14.3 ± 0.2	13.5 ± 0.3 g/dl	**<.05**
	post-bypass initiation meas.	10.9 ± 0.2	9.9 ± 0.3 g/dl	**<.05**

BMI-body mass index, BSA-body surface area, CPB-cardiopulmonary bypass, meas- measured, calc- calculated

### Tissue oxygenation index

**Figure 1.**
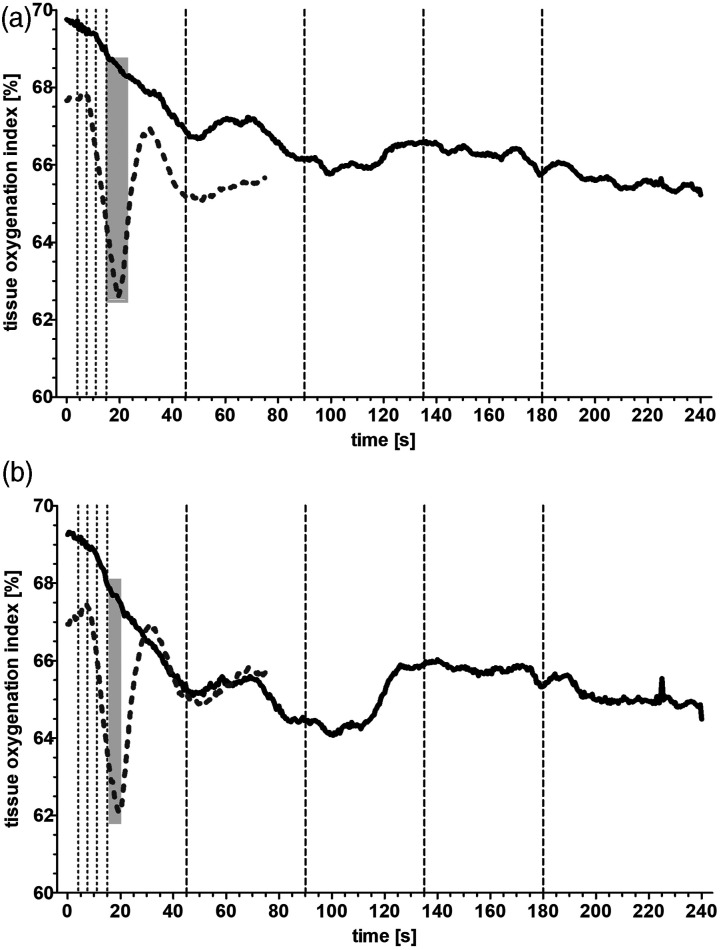
(a). Tissue oxygenation index curve of NIRS-values measured over the left forehead. Displayed are the mean values of the slow initiated CPB (Group A, black line) and of the fast initiated CPB (Group B, gray dotted line). Vertical interrupted lines correspond to 25%, 50%, 75%, and 100% of target flow rate in the respective groups. Light gray bar highlights a significant difference between the curves (*p* < .05). (b). Corresponding graph over the right forehead.

The initiation of the HLM led to a significant decline in the oxygenation index of the measurement sites reaching a stable level after 60 s at the target flow rate in both groups (Figure 1), but the fast initiation exemplified a two-phased curve with a steep decline to a nadir and a subsequent steep incline to a vertex of 67.6 ± 1.6% (left side) and 68.6 ± 1.4% (right) at 37 ± 3 s (left) and 31 ± 2 s (right) respectively after leveling off to a plateau. In the relative comparison of the curves the difference in oxygenation index reached a significant level right after the fast initiated HLMs had their target flow rate in a time frame of 15.5 s–22.5 s (Figure 1(a), gray bar; *p* < .05) and 18 s–20 s (Figure 1(b), gray bar; *p* < .05).

**Figure 2.**
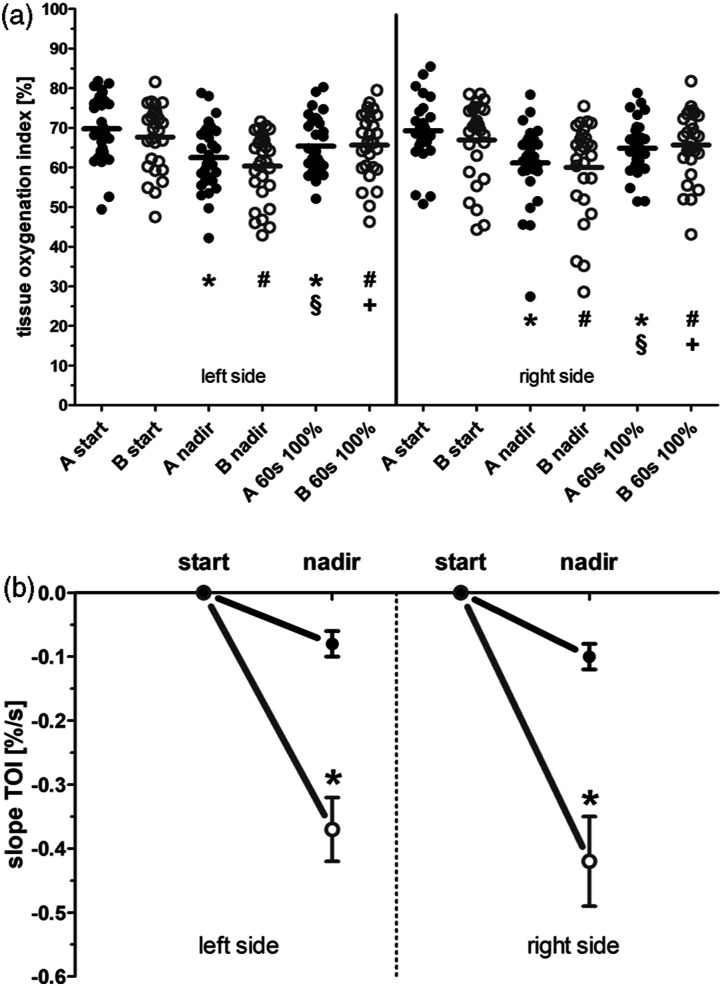
(a). Tissue oxygenation index readings of the left and right forehead at start of CPB, nadir and 60 s after the target flow rate was reached. Displayed are the single values and the mean (cross line) of the slow initiated CPB (Group A, black dots) and of the fast initiated CPB (Group B, gray open circles). * *p* < .05 vs. A start, § *p* < .05 vs. A nadir. # *p* < .05 vs B start, + *p* < .05 vs. B nadir. (b). Rate of change in tissue oxygenation index of the left and right forehead expressed as individual slope calculations from start to nadir reading in Group A (black dots, black line) and Group B (gray open circle, gray line). Data values are means ± standard error. TOI-tissue oxygenation index.

At the start of CPB (Figure 2(a)) there was no significant difference in the TOI with lower values in Group B (left side – 67.7 ± 1.6%; right side – 66.9 ± 1.8%; *n* = 29) than in Group A (left side—69.8 ± 1.5%; right side—69.3 ± 1.4%; *n* = 31, ns) as well as there was no difference in the absolute values in both groups (A: left side—65.4 ± 1.3%; right side—64.9 ± 1.2%; *n* = 31; B: left side—65.7 ± 1.5%; right side—65.7 ± 1.6%; *n* = 29, ns) after 60 s at the target flow rate. Looking at the kinetic the decrease in TOI was more pronounced in Group B with a steep decline to a nadir of 60.4 ± 1.6% (left side) and 60.0 ± 2.3% (right) at 26 ± 3 s (left) and 21 ± 2 s (right), respectively, after starting the CPB with a higher rate of change (Figure 2(b)) in oxygenation index (left side: −0.37 ± 0.05%/s; right side: −0.42 ± 0.07%/s). The slower initiation of the CPB produced a significant lower rate of change (Figure 2(b)) with −0.08 ± 0.02%/s (left side; *p* < .05) and −0.10 ± 0.02%/s (right side; *p* < .05) in the oxygenation index when compared to the group with the fast initiation. Despite this rate of change the absolute nadir values of Group A (left side—62.5 ± 1.5%; right side—61.1 ± 1.7%) are not different than those of Group B (Figure 2(a)).

When looking at the accepted critical values of cerebral oximetry for probable ischemic events, Group A exhibited 1(left)/2(right) TOI nadir readings under 50%, in contrast to 6(left)/5(right) TOI nadir readings in Group B. A decline over 20% to baseline TOI reading was detected in 1(left)/4(right) patients in Group A, in contrast to 4(left)/5(right) patient in Group B. A decline of over 80% was not seen in any group (data not displayed).

### Normalized tissue hemoglobin index

**Figure 3.**
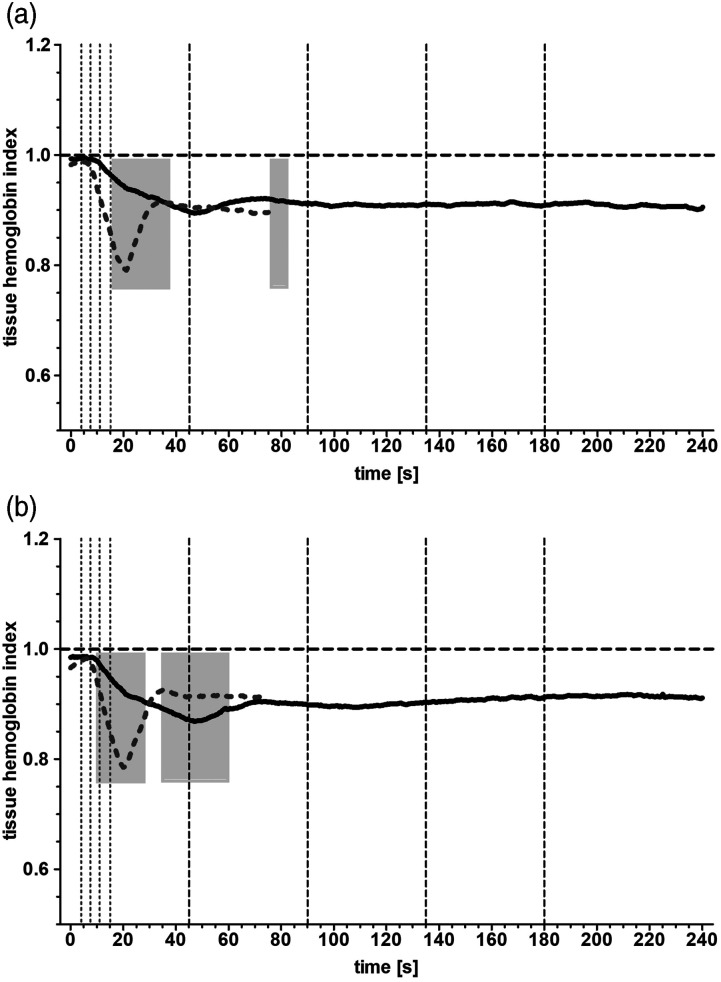
(a). Normalized tissue hemoglobin index curve measured over the left forehead. Displayed are the mean values of the slow initiated CPB (Group A, black line) and of the fast initiated CPB (Group B, gray dotted line). Vertical interrupted lines correspond to 25%, 50%, 75%, and 100% of target flow rate in the respective groups. Light gray bars highlight a significant difference between the curves (*p* < .05). (b) Corresponding graph over the right forehead.

The curves of the hemoglobin index showed a hemodilution curve similar to their respective tissue oxygenation curves (Figure 3). In either group, the dilution of the patients’ blood is about 10% with an index of 0.90 ± 0.01 (Figure 3(a): left side, both groups) and 0.91 ± 0.01 (Figure 3(b): right side, both groups) and the end of the measurement period. Fast initiation of CPB led to a rapid dilution of blood reaching significant difference between 8.5 s to 29 s (left side) and 9.5 s–27 s (right side) to Group A (Figure 3, first gray bars; *p* < .05). After the hemodilution was complete in Group B, there was a second time frame (left side: 68.5 s–75 s; right side: 34 s–60.5 s) where the curve was significantly different to that of Group A (Figure 3, second gray bars; *p* < .05).

## Discussion

Our study demonstrated for the first time the different kinetics of change in regional tissue oxygenation during a slow and fast initiation of CPB to reach the prospected target flow. This change in tissue oxygenation was congruent with the negative change in the normalized tissue hemoglobin index, revealing the direct impact of hemodilution on tissue oxygenation measured by the NIRS method, a well-documented tool in effectively monitoring the patient’s cerebral oxygenation during CPB.^8^

The effect of hemodilution of the HLM is thought to be greatest during initiation as the priming volume of the machine is abruptly pumped into the blood circulation. Subsequently, the most significant negative change in mean cerebral oxygenation happens during this initiation period as described by Ševerdija.^9^ Furthermore, overall reduced cerebral oxygenation throughout the entire bypass period is well described.^9–11^ In this study we decided to use the near infrared spectroscopy by Hamamatsu Photonics as it offers sampling intervals up to 0.05 s as compared to 5 s (INVOS™; Medtronic, USA) or 1.5 s (EQUANOX™; Nonin Medical Inc, USA), a clear advantage for the fast changes in oxygenation in our study. At a set sampling rate of 0.5 s, we were able to detect the two-phased curve (steep decline followed by a steep incline) in cerebral oxygenation when the HLM was ramped up to 100% in 15 s, contrasting with the more linear decline in oxygenation when a ramp-up time of 180 s was used. Despite this difference in the rate of change, the absolute values of the impact on tissue oxygenation showed no difference in nadir or at 60 s after reaching the 100% of the target flow rate of the HLM, explainable through the same dilutional volume of the priming solution in both groups. Likewise, the difference in left- and right-sided obtained NIRS values, though not significant, is probably due to either a slightly more diluted blood entering the right head vessels as they are anatomically nearer to the delivery cannula of the bypass or through the produced jet aligned to the left carotid artery by the direction of cannula placement. Higher readings in left versus right oxygenation during CPB is described in the literature,^12^ and our pooled mean value of TOI at the time point of completed hemodilution with a value of 65.4 ± 0.7% is similar to that of a recent meta-analysis.^12^ As severe hemodilution alters cerebral oxygenation and higher blood pump flow rates are needed to achieve adequate cerebral oxygen supply on CBP,^13^ we designed the study in exclusion of anemic patients to achieve a reliable post-dilutional hemoglobin level of equal or more than 10 g/dl in both groups. It has been shown that avoidance of an 80% decline to preoperative TOI values or absolute TOI values under 50% will eventually help to reduce postoperative neurological sequalae and morbidity after CPB.^14^ Further evidence can be derived from the balloon protection method in carotid artery stenting, where the cutoff TOI in detecting ischemic intolerance was 50% or an 80% TOI change rate as well.^15^ Although one can assume that our fast- and slow-initiation CPB protocols were both safe methods for patients when looking at the grouped numerical results, it is obvious that with the higher rate of critical values (21% vs. 6%) in the fast group, one might think differently when considering individual patient safety. Even though it is still not known if fast initiation of the CBP negatively influences clinical outcomes in patients with impaired cerebral blood supply (anemia, carotid stenosis, etc.), it seems plausible looking at our results. So far there are no known studies looking at the impact of different CPB management strategies in these specific patient groups despite known negative impacts of the CPB including enhanced shear stress, gaseous microemboli or reactive alterations in cerebral blood flow.^16–18^

Despite the limitation in our study of lower hematocrit and hemoglobin levels in the fast-initiation group and no postoperative patient follow-up, the authors believe that this study adds important new evidence to the cardiovascular perfusion field. As the hemodilution curve in the slow initiated, CPB group leveled off to stable values between 45 and 90 s (25–50% of target flow) without further changes afterward, we assume that a reasonable initiation time lies in between those limits, knowing that at this point it is being a very hypothetically assumption. In conclusion, we believe that it is safer to slower ramp-up the HLM to 100% of target flow rate in order to avoid critical phases of cerebral ischemia especially if anemia or carotid stenosis is present, and therefore propose an initiation time of at least 90 s in all CPBs.
